# Gold–copper oxide core–shell plasmonic nanoparticles: the effect of pH on shell stability and mechanistic insights into shell formation†

**DOI:** 10.1039/d3na01000g

**Published:** 2024-04-15

**Authors:** Stephen F. Bartolucci, Asher C. Leff, Joshua A. Maurer

**Affiliations:** a US Army Combat Capabilities Development Command Armaments Center Watervliet NY 12189 USA stephen.f.bartolucci.civ@army.mil joshua.a.maurer4.civ@army.mil; b US Army Research Directorate, Combat Capabilities Development Command, Army Research Laboratory Adelphi MD 20783 USA; c General Technical Services, LLC, Wall NJ 07727 USA

## Abstract

Plasmonic nanoparticles play an important role in applications for chemical sensing, catalysis, medicine, and biosensing. The localized surface plasmon resonance (LSPR) of a nanoparticle is determined by factors such as size, shape, and the local dielectric environment. Here, we report a simple colloidal synthesis method to create core–shell plasmonic nanoparticles with a gold core and a copper oxide (Cu_2_O) shell. The gold cores are particles of various shapes and sizes, including nanorods, nanobipyramids, and nanoshells, and the Cu_2_O shell is on the order of 30–40 nm thick. The growth of the oxide shell red shifts the plasmonic absorption of the gold core particle by up to 250 nanometers, resulting in a particle that can absorb into the near-infrared (NIR). Additionally, we report the unique ability to immediately remove and regrow the oxide shell by simple changes to the solution pH. We demonstrate the repeated dissolution and nucleation of the oxide shell through the addition of an acid and a base, respectively. The process is confirmed by characterization using Ultraviolet-Visible-Near-IR (UV-Vis-NIR) spectroscopy and electron microscopy of the particles. After several iterations of this process, we report the formation of large Cu_2_O spheres, where Cu_2_O nucleation on other Cu_2_O particles is favored over the gold nanoparticles. In addition, we provide insight into the role of ligands in shell formation.

## Abstract 2

Gold–Copper oxide core–shell nanoparticles of various shapes were synthesized, and the stability of the oxide shell was studied during pH cycling as well as the importance of ligands in shell formation.

## Graphical abstract

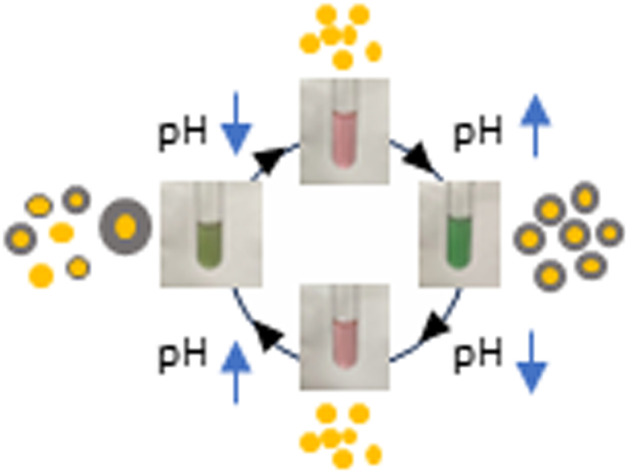

## Introduction

Localized surface plasmon resonance (LSPR) is the coherent oscillation of conduction electrons in subwavelength metallic nanoparticles when coupled to light. The LSPR is affected by the particle composition, size, shape, and surrounding material. This optical response of the surface plasmon can be utilized in a number of applications, including sensing, photocatalysis, photovoltaics, and biomedical applications.^1–4^ Simple colloidal-based aqueous synthesis of metallic nanoparticles has allowed researchers to tailor the size and shape of the particles for specific applications. In the last couple of decades, there has been extensive research in the synthesis of nanoparticles with various shapes, including round particles, nanorods, nanostars, nanocubes, nanobipyramids, and hollow spheres, just to name a few. Noble metals, such as gold and silver, have strong plasmonic properties and tend to have a narrow line shape that is dependent on particle size.^5^ Additionally, gold nanoparticles can exhibit significant plasmonic shifts upon changes in their local environment. This has led to the study of core–shell particles, where the core particle is a noble metal such as gold or silver and the shell material is another metal, or a metal oxide. Many types of metal oxides have been studied, including titanium dioxide (TiO_2_), silicon dioxide (SiO_2_), tin oxide (SnO_2_), zinc oxide (ZnO) and copper oxide (Cu_2_O).^6^ These materials have been explored for use in gas sensing and photocatalysis. In photocatalysis, it has been shown that the plasmon energy can be transferred from the metal to the semiconductor oxide shell *via* plasmon-induced resonant energy transfer, generating electron–hole pairs and photocatalytic enhancement.^7^

Cu_2_O (cuprous oxide) is a p-type semiconductor with a lattice mismatch of 4.5% with gold (Au) and has been reported to have a refractive index of 2.7 at wavelengths above 600 nm.^8^ When coupled to a noble metal, this high refractive index may lead to large shifts in the plasmon extinction spectra. The thickness of the Cu_2_O shell can be varied to tune the extinction spectra of the particles.^9^ Gold core particles of certain shapes with the combination of an oxide shell can shift the extinction spectra of the particle into the near-infrared. Huang and coworkers demonstrated the photothermal effects from Au-Cu_2_O core–shell particle with near-infrared absorption tunability.^10^ The near-infrared spectrum is biologically important and has uses in both therapeutic and imaging applications in tissue because biological molecules do not have significant background absorption in this wavelength range.^11^

Cuprous oxide shell growth on gold has been of interest for various applications, such as sensing^12^ and photocatalysis.^13^ Huang and coworkers have shown the growth of Cu_2_O on gold nanoparticles of various shapes in aqueous solution^14^ and have performed several studies on the facet-dependent optical properties of Au-Cu_2_O core–shell nanocrystals.^15,16^ While many studies have shown application of these particles and discussed the optical properties with respect to plasmonic absorption, there have been few studies on the precise growth mechanisms, and even fewer on the chemical stability of the Au-Cu_2_O core–shell particles. Zhu *et al.* studied the stability of these particles in water and ethanol mixtures and found that mixtures with a larger amount of water were more likely to reduce the stability of the Au-Cu_2_O particle.^17^ It is known that the pH of the solution can affect copper oxide stability.^18,19^ However, it has not been demonstrated that *in situ* changes to the pH can rapidly dissolve and reform the shell material on a gold nanoparticle. In this work, we show the ability to use pH as a method to do this cycling and reveal the effect it has on the structure and quality of the oxide shell, as well as the formation of secondary nanoparticles. We also demonstrate the importance of ligands in the formation of the copper oxide shell on the gold nanoparticles.

## Experimental

### Materials and synthesis

**Fig. 1:**
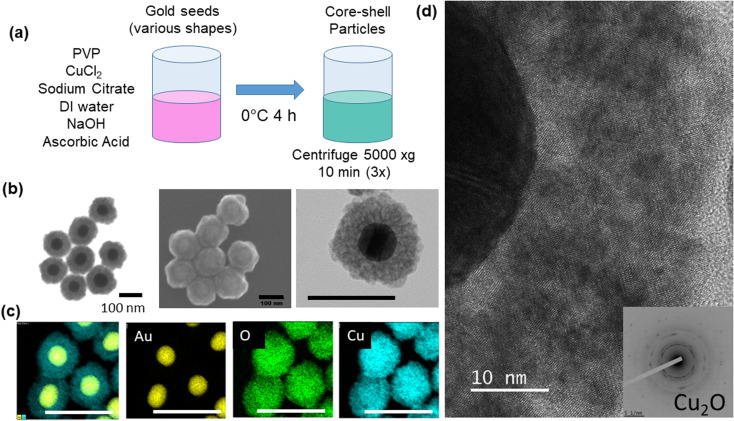
(a) Core-shell particle synthesis method (b) electron microscopy of 50 nm Au@Cu_2_O (scale bars are 100 nm) and (c) energy dispersive spectroscopy of Au@Cu_2_O (scale bars are 250 nm) (d) TEM and SAED of Cu_2_O shell.

Core-shell particles were synthesized using colloidal methods based on Liu *et al.*^9^ Gold spheres with 50 nm diameter (STREM, 50 μg mL^−1^, citrate stabilized), gold nanorods (25 nm diameter and 90 nm length, Aspect Ratio (AR) = 3.6, 50 μg mL^−1^), larger gold nanorods (25 nm diameter and 102 nm length, AR = 4.1), gold nanobipyramids (40 nm diameter with 140 nm length), and 50 nm gold nanoshells (Nanopartz), respectively, in the amount of 1.25 mL were added to 0.125 mL of 10 mM polyvinylpyrrolidone (PVP) (TCI, 10 kDa MW), 0.025 mL of 0.1 M trisodium citrate dihydrate (VWR) and 0.063 mL of 20 mM copper chloride, CuCl_2_ (MilliporeSigma). In some experiments, CuCl (MilliporeSigma) was used in place of CuCl_2_ and in other experiments dehydroascorbic acid (MilliporeSigma) was used in place of ascorbic acid at the same molar concentrations, respectively. The mixture was diluted to 3.125 mL with deionized water (18.2 MΩ cm) and cooled to approximately 0 °C in an ice bath. The mixture was then blended with 0.035 mL of 1 M sodium hydroxide (NaOH) and 0.25 mL of fresh 0.1 M ascorbic acid (VWR), mixed by one inversion, and allowed to sit for 4 hours. The pH of the solution was measured (Mettler Toledo SevenExcellence™) at the beginning (0 h) and end (4 h) of the growth period, at 20, 26, and 44 h after start of growth, and after additions of 40 μl of NaOH (5 M) and hydrochloric acid (HCl) (5 M) in order to study the effect of pH changes on the shell structure. The volume of the 40 μL addition of acid or base was adjusted to account for the decreased volume of solution after each round, due to sample aliquots being removed. The adjustment was made so that the same volume percentage (0.8 vol%) was added for each round. Particles were centrifuged at 5000×*g* for 10 minutes and washed in deionized (DI) water three times prior to an analysis (Fig. 1a). In one experiment, gold nanoparticles (1.25 mL) were treated with 11.3 μL 1-propanethiol (TCI) for 1.5 h.

### Characterization

Particles were imaged in STEM (scanning transmission electron microscopy) mode using an FEI 600i Helios NanoLab™ Dual-Beam electron microscope at 30 kV after drop casting on a nickel formvar/carbon square 300 mesh transmission electron microscopy (TEM) grid (Electron Microscopy Sciences). High-resolution imaging, energy dispersive spectroscopy (EDS), and selected area electron diffraction (SAED) was conducted using a JEOL ARM200F probe-corrected TEM operated at 200 keV. Ultraviolet-Visible-Near-IR spectroscopy (UV-Vis-NIR) spectra were collected using an Agilent Cary 7000 Series UV-Vis-NIR Spectrophotometer in a quartz cuvette. Data were baselined and normalized to unity for clarity. Zeta Potential measurements were conducted on a Brookhaven NanoBrook Zeta PALSon dilute solutions. Photoluminescence (PL) measurements (350–850 nm) were obtained with a Horiba Fluorolog^®^-3 Spectrofluorometer equipped with a detector (190–850 nm) using an excitation scan from 300–500 nm and an emission scan from 340–850 nm (integration time: 0.3 s, excitation slit: 10 nm, emission slit: 10 nm, increment: 10 nm).

## Results and discussion

**Fig. 2:**
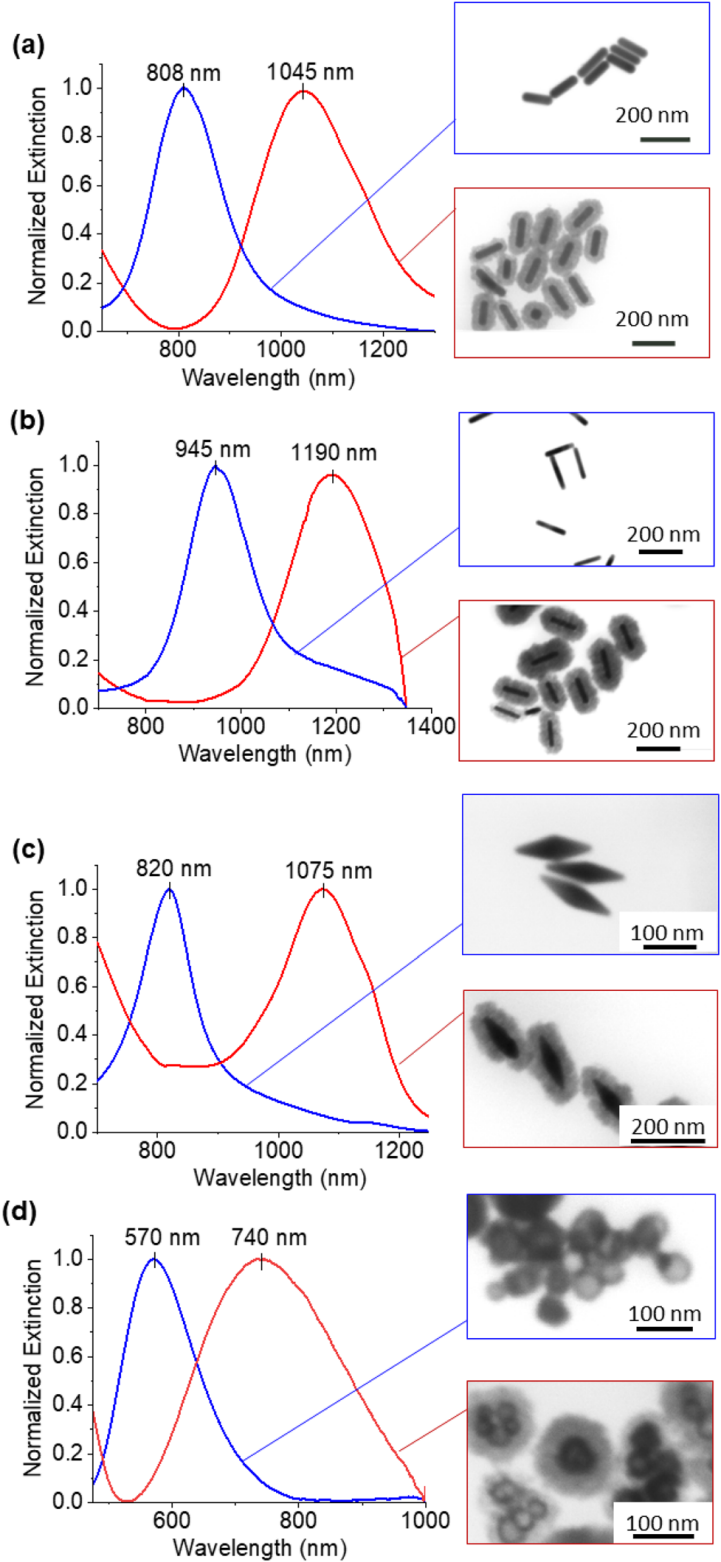
UV-Vis-NIR extinction spectra for gold nanorods (a) AR = 3.6 and (b) AR = 4.1 (c) nanobipyramids and (d) nanoshells.

The core–shell particles created with spherical 50 nm gold cores are shown on Fig. 1b, with the EDS (Fig. 1c) spectra confirming the gold and copper oxide core–shell structure. For the growth conditions outlined in the Experimental section, the typical Cu_2_O shell thickness is between 30–40 nm (Table S1†), as measured by STEM. The shells consisted of nanocrystalline Cu_2_O, which heterogeneously nucleates or adsorbs onto the gold core particle, as confirmed by TEM and SAED shown in Fig. 1d and S1.† An extinction peak of 533 nm for the gold seeds is red shifted to 622 nm with the addition of the oxide shell. To explore the formation of the Cu_2_O shell on shapes other than spherical particles, gold nanorods with different aspect ratios, nanoshells, and nanobipyramids were used as the core materials, and the results are shown in Fig. 2. Gold nanorods and nanobipyramids have two plasmon peaks, one associated with electron resonance in the longitudinal direction, and one in the transverse direction. The data presented in Fig. 2 for these particles focuses on the longitudinal peak; however, the full spectrum above 350 nm can be seen in Fig. S2.† Gold nanorods (25 nm diameter and 90 nm length, AR = 3.6) display an extinction peak at 808 nm for the longitudinal plasmon (Fig. 2a). After the growth of the Cu_2_O shell, the extinction peak red shifts to 1045 nm, a shift of 237 nm. Likewise, the gold nanorods (25 nm diameter and 102 nm length, AR = 4.1) had an initial extinction peak at 945 nm and red shift with the Cu_2_O shell to 1190 nm, a shift of 245 nm (Fig. 2b). The nanobipyramids red shift from 820 nm to 1075 nm, a shift of 255 nm (Fig. 2c) and the nanoshells red shift from 570 nm to 740 nm, a shift of 170 nm (Fig. 2d). The nanoshells have an inhomogeneous distribution of starting shell sizes, and, additionally, some of the core–shell particles incorporate more than one core in the shell, resulting in the largest full width at half maximum (FWHM) for the core–shell particles. In the case of all the particles, the core–shell structure spectra are slightly broadened, likely a result of variations in the oxide thickness. A summary of the peak data with FWHM data quantifying the broadening, along with oxide shell thicknesses, is shown in Table S1.† The oxide shell on the nanorods and nanobipyramids resulted in extinction well into the near-infrared. The large shift in plasmon extinction can be explained by the refractive index sensitivity of shapes such as rods and bipyramids. The plasmon shift for shapes with high refractive index sensitivities increases more rapidly with an increase in the refractive index of the surrounding dielectric medium. In this case, the index increases from 1.3334 (water) to 2.7 (cuprous oxide). In addition to properties of shape like aspect ratio, index sensitivity is further increased by sharper apexes, such as those found on the bipyramids. The results here are consistent with a study by Wang and coworkers,^20^ where they studied the index sensitivities of gold nanorods and bipyramids by increasing the index of refraction from pure water to a water/glycerol mixture.

Cu_2_O is a semiconductor with a bandgap of 2.17 eV and can photoluminescence when irradiated with light of a higher energy than the bandgap. While it is beyond the scope of this work, it has been shown that there can be a coupling between plasmons and photoluminescent (PL) emission^21^ and enhanced photocatalytic activity, where concentrated energy contained in localized plasmonic oscillations is transferred to the semiconductor, inducing charge separation in the semiconductor.^22^ The particles were characterized for PL excitation and emission, and the measurements are shown in Fig. S3† for various particle shapes with oxide shells. The excitation wavelengths for all the particles fall between 300–320 nm and PL is observed by the particles. Emission wavelengths varied, depending on particle shape, between 460 and 850 nm.

**Fig. 3:**
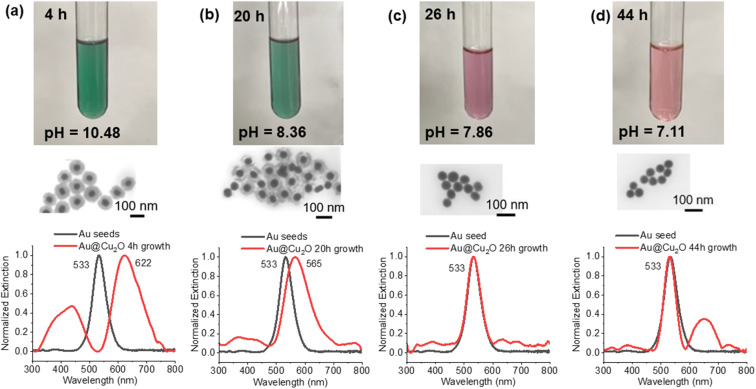
Process of the core–shell particles slowly losing the Cu_2_O shell after growth. Samples were taken (a) 4 hours, (b) 20 hours, (c) 26 hours and (d) 44 hours after the start of particle growth.

For the various core–shell particles, it was observed that unwashed particles reverted to the gold core particles after approximately 24–48 hours of storage at 4 °C. Samples were analysed using UV-vis and electron microscopy, and it was determined that the Cu_2_O shell had disappeared. Particles that were washed and stored in pure DI or in ethanol retained the core–shell structure indefinitely. To determine the cause of the oxide shell dissolution, experiments were performed to measure the pH of the solution as a function of time and the morphology of the particles using UV-vis and STEM. The results of these measurements are shown in Fig. 3. At 0 h, the materials were mixed to start the growth of the core–shell particles, and at 4 h, the growth of the core–shell particles was complete. The pH was measured to be 11.28 at 0 h and 10.48 at 4 h (Fig. S4†). Cu_2_O shells were clearly formed, and the particles had an extinction peak at 622 nm with smaller peaks around 385, 440 nm, and 740 nm. The 622 nm peak is from the gold plasmon that is red shifted due to the Cu_2_O shell. At longer times, 20 h after start of growth, the pH dropped to 8.36 and the Cu_2_O shell was still present on most gold particles. There was clear degradation of the shell in the STEM and a corresponding blueshift of the main gold extinction spectrum to 565 nm. The dual peaks around 370 and 430 nm also decreased in intensity, with the 370 nm peak being stronger than the 430 nm peak and a faint peak at 765 nm. At 26 h, the solution clearly turned from a green color to a purple/pink color, and the pH dropped to 7.86. STEM images showed nearly all gold particles with no Cu_2_O shell present, and this was confirmed by UV-vis, which had the same strong extinction at 533 nm. Small peaks at 385, 635, 680, 735, and 760 nm were observed. Further aging to 44 h resulted in a pink solution with a pH of 7.11. STEM images showed all gold particles and UV-vis with a main peak at 533 nm and a peak at 650 nm, along with smaller peaks at 330 and 385 nm and one at 738 nm. The dissolution of the Cu_2_O shell appeared to occur as the pH of the solution dropped.

**Fig. 4:**
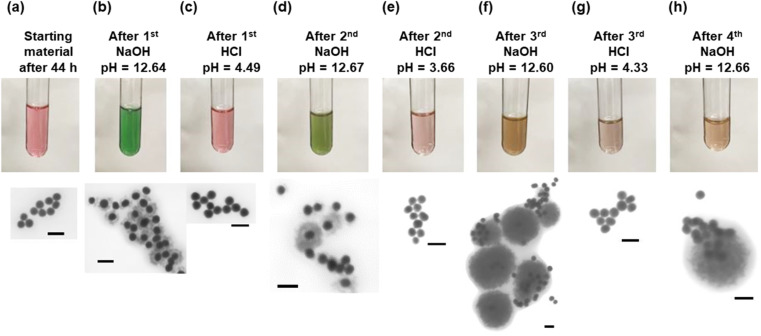
The (a) starting particles 44 h after the start of Au-Cu_2_O core–shell growth had reverted to the gold core material, (b) NaOH addition quickly increases the pH and formation of Cu_2_O shell. Subsequent additions of (c) HCl lowers the pH and removal of the shell. The second cycle of (d) NaOH shows some shell formation with some thicker shells on fewer gold particles, and (e) HCl additions again show reversal to gold core particles without shells. The third cycle (f) NaOH shows many gold particles without shells and the formation of large Cu_2_O particles approximately 400 nm in diameter and (g) the third cycle of HCl reverting all particles back to gold cores. The fourth cycle (h) of NaOH showed mostly gold particles and an occasional large Cu_2_O particle (scale bars are 100 nm).

Since the dissolution of the oxide shell appeared to be correlated to the pH of the particle solution, experiments were performed to change the pH and observe the effect on the shell. The results of these experiments are shown in Fig. 4. The particle solution aged for 44 h was the starting material for the cycling of the core–shell particles. As was observed in Fig. 3, the solution had turned pink and consisted of only gold core particles (Fig. 4a). Upon addition of the first NaOH, the pH increased to 12.64, and the solution turned green in approximately 1 minute as a result of the formation of the Cu_2_O shell around the gold core. STEM confirmed the presence of the shell, as seen in Fig. 4b. Upon addition of the HCl, the solution with the core–shell particles immediately turned pink as the pH dropped to 4.49. STEM analysis showed only the presence of the gold cores. After the second NaOH addition to the pink solution, it again turned green in about 1 minute. The green color was a different shade than it was after the first addition of NaOH, and STEM analysis showed a mixture of gold core particles and gold core particles with a Cu_2_O shell. The shells appear to be thicker than the shells present after the previous NaOH addition. Again, the second addition of HCl resulted in a pink solution with a pH of 3.66, and the STEM showed gold core particles. An interesting change occurred after the third addition of NaOH. The solution, which again has a pH of 12.60, turned an amber color. This color is consistent with aqueous solutions containing Cu_2_O particles under 425 nm in diameter, as reported by Halas and co-workers.^23^ STEM analysis showed the presence of large particles with a diameter of approximately 400 nm. While some of the particles appeared to have a gold core associated with them, most of them did not have a gold core. Additionally, it was not clear whether those gold particles were in the core, or merely adjacent to the large oxide particles. The third HCl addition resulted in a solution pH of 4.33 and only gold particles visible. The final NaOH addition had a pH of 12.66 and mostly gold particles and large Cu_2_O particles, as observed in the previous NaOH addition. EDS confirmed that the particles are copper oxide (Fig. S5†).

Cu_2_O nanospheres similar to the particles observed after the third and fourth cycles of NaOH have been reported previously. With the effect of PVP, Zhang *et al.* showed primary Cu_2_O nanocrystals prefer to aggregate into spherical monodisperse nanospheres.^24^ Zhang and Wang synthesized Cu_2_O nanospheres and hollow nanospheres with an outer diameter around 420 nm at room temperature, with PVP believed to play a crucial role in mediating the aggregation and packing of the Cu_2_O nuclei into solid spheres.^25^ Yang *et al.* also reported similar sized Cu_2_O hollow spheres.^26^ The Cu_2_O nanospheres were only observed after the third NaOH addition, indicating that the Cu_2_O nanocrystals prefer to nucleate on other Cu_2_O nanocrystals rather than the gold particles. The NaOH/HCl cycling seems to play a role in making the Cu_2_O nanocrystals form the nanospheres instead of nucleating on gold, but the cause of it is unclear. It could be due to the degradation of the citrate ligands on the gold nanoparticles from the HCl/NaOH cycling. Zeta potential measurements that we conducted showed that HCl resulted in a strong reduction in the negative potential of the gold nanoparticles (from −22.89 mV to −0.38 mV). If the negatively charged ligands were degraded or removed, the positively charged copper ions would not be attracted to the gold seed particles and would likely form larger Cu_2_O particles, as we observed.^27^ To prove this hypothesis, the starting gold nanospheres were treated with 1-propanethiol to exchange the negative citrate ligands with the neutral thiol ligands. Using the same growth procedures and conditions, except for using the gold nanoparticles with thiol ligands and no addition of citrate, core–shell particles were not observed to form. STEM analysis showed only gold seeds and particles that contained copper (Cu) and oxygen (O) by EDS (Fig. S6†). In the absence of gold nanoparticles, the original growth solution will form small Cu_2_O particles (20 nm) with an extinction peak at 350 nm, consistent with small crystals of Cu_2_O (Fig. S7†).

**Fig. 5:**
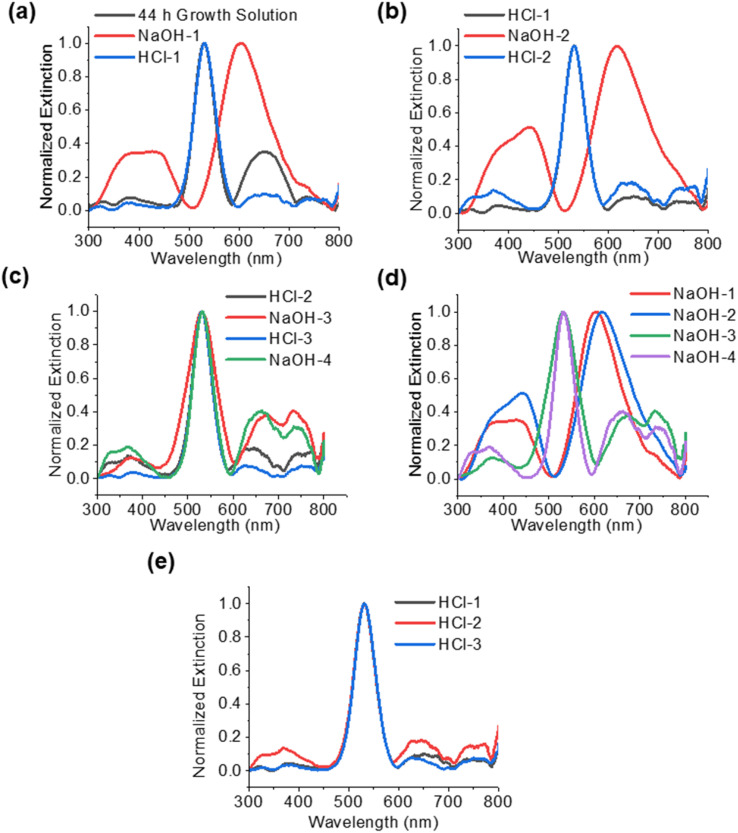
UV-vis spectra of the NaOH/HCl cycled particles. (a) The starting solution is the material 44 h after start of growth. NaOH-1 and HCl-1 is the solution after the first addition of NaOH and subsequent first addition of HCl, respectively. (b) Spectra comparing the solution after the first HCl addition to the subsequent addition of the second NaOH and then the second HCl. (c) Spectra comparing the material after the second HCl addition to the subsequent third NaOH, third HCl, and fourth NaOH additions. (d) Spectra of all the solutions after NaOH additions and (e) spectra of all the solutions after HCl additions.

The UV-vis spectra are a result of the structures and materials present in each phase of the experiment, based on the conditions. The spectra can be a combination of the absorption of the gold particles, the absorption and scattering of the core–shell structures, interband transitions in the Cu_2_O, and scattering by large Cu_2_O nanoparticles. Fig. 5 shows the spectra for the solutions after each treatment with NaOH and HCl, as described in the previous section. In Fig. 5a, the particles after 44 h have a strong extinction peak at 533 nm, indicative of only gold core nanoparticles. After the addition of NaOH (NaOH-1), there is a red shift in the main extinction peak to 605 nm. This is slightly blue shifted from the original core–shell particles, which had an extinction peak of 622 nm (Fig. 2a) after 4 h of growth. Additionally, there is a large peak that consists of two convoluted peaks at 440 and 385 nm, which were also seen after the original 4 h growth of the particles (Fig. 3a). These are attributed to the Cu_2_O nanocrystals in the shell, since these peaks are only observed in particles that have a shell. In the samples 20 h after growth and later (Fig. 3), these peaks greatly decrease in relative intensity as the shell is dissolved. Borgohain *et al.* reported absorption at 448 nm for Cu_2_O nanocrystals that were 8 nm in diameter.^28^ According to our TEM analysis (Fig. 1d), the nanocrystals in our shells are below 10 nm in size and correspond well to the reported UV-vis absorptions in this range. After addition of HCl (HCl-1), the extinction spectra revert to the starting particles (Au@Cu_2_O after 44 h) with a strong gold plasmon peak at 533 nm. The peaks at 385, 440, and 650 nm become relatively weak. In Fig. 5b, the second addition of NaOH (NaOH-2) again results in a red shifted main extinction peak from 533 to 618 nm, as a result of Cu_2_O shells forming on the gold nanoparticles. In addition, the peak at around 440 nm is stronger relative to the 380 nm peak and is due to the extinction by Cu_2_O nanocrystals in the shell.^23^ In fact, we only observe the peak around 440 nm when a Cu_2_O shell is present. After the second HCl (Fig. 5b), the main peak reverts to 533 nm, as expected, and the 440 nm peak disappears. Smaller peaks below 400 nm can be seen, similar to what was observed after the first HCl treatment. In Fig. 5c, we see a strong 533 nm peak even after the addition of the third and fourth cycles of NaOH (NaOH-3 and NaOH-4). This is consistent with the STEM in Fig. 4 which showed gold core nanoparticles and the formation of the Cu_2_O nanospheres. In addition to the absorption by the gold nanoparticles, the Cu_2_O nanospheres and nanoparticles will contribute features to the extinction spectra. Extinction spectra showed multiple features, largely due to light scattering at the Mie resonances of the particles. Bimodal peaks are seen around 675 nm and 738 nm. Wang and co-workers^25^ synthesized Cu_2_O nanospheres and nanoshells with 420 nm diameter and they observed broad extinction above 600 nm, and as the shell thickness decreased, interband transitions of Cu_2_O below 400 nm dominated, which is what could be observed in Fig. 5d for the last two cycles of NaOH treatment. Additionally, Yang *et al.* synthesized hollow nanospheres 300–600 nm in diameter with a broad absorption around 505 nm.^26^ Zhang^24^ also showed nanospheres with broad scattering around 520 nm. Yin *et al.* reported^29^ small Cu_2_O nanocrystals show absorption for 2–10 nm crystals from 609 nm to 659 nm, respectively. The observed peaks around 650 nm could be attributed to absorption by small crystals in this size range. The spectra after HCl additions are summarized in Fig. 5e and are consistent.

While we observed the dissolution of the Cu_2_O shell with lowering pH, the cause of this drop in pH was not established. It is possible that carbon dioxide (CO_2_) from the air is lowering the pH of the growth solution. To test this, a vial of growth solution was bubbled with Argon and capped. The solution stayed green (core–shell particles) for over 5 days, and then slowly turned pink, as air leaked into the vial and the oxide shell dissolved. In another experiment, the green growth solution with core–shell particles was bubbled for 2 minutes with CO_2_ gas and it turned purple during bubbling. That material was viewed by STEM, and it showed copper oxide spheres next to gold spheres, but not a full core–shell morphology (Fig. S8†). This solution then turned pink within 48 h. These experiments seem to indicate that the drop in pH during the first 24 h that we observed was due to the absorption of CO_2_ from the atmosphere, which led to the dissolution of the oxide shell.

When ascorbic acid was not added to the growth solution, an oxide shell did not form. Similarly, an oxide shell did not form in the absence of NaOH, as both appear to be necessary for oxide shell growth. Ascorbic acid reduces Cu^2+^ to Cu^1+^, likely from copper(ii) hydroxide. An additional growth experiment was performed using CuCl, which also resulted in the growth of Cu_2_O shell (Fig. S9†). In this case, the Cu is already in the +1 oxidation state and does not need to be reduced. However, we found that the reaction did not proceed without the addition of the ascorbic acid.

Further, a growth solution was prepared with 0.1 M dehydroascorbic acid (DHA) in place of the ascorbic acid, for both CuCl_2_ and CuCl, respectively, and only the growth solution with CuCl formed core–shell particles (Fig. S10†). This demonstrates that the DHA molecule must play a role as a ligand in the formation of the shell. In the case of the copper(ii) chloride, the ascorbic acid reduces the copper(ii) to copper(i) and oxidizes to DHA.^30^ This oxidation provides the DHA to serve as a ligand. In the case of copper(i) chloride, reduction is not needed, so DHA can be added as a ligand. For copper(i) with ascorbic acid, it is unclear if ascorbic acid or DHA produced from air oxidation is serving as the ligand. Attempts to use molecules with similar structural features to DHA in place of DHA or ascorbic acid with copper(i) chloride did not yield core–shell particles. These alternative ligands included sucrose and 1–2 butanediol. The DHA growth solution quickly turned from green to pink in 3.5 h after the start of growth. Subsequently, 40 μL of 1 M NaOH was added to the pink solution, and it turned green again with core–shell nanoparticles.

A cycling experiment was conducted on washed particles, and the particles did not cycle like the unwashed particles. The addition of the HCl turned the green solution pink, indicating dissolution of the shell, but additions of NaOH did not cycle the particles back to green core–shell particles. STEM analysis only showed the gold seeds. The lack of cycling is likely due to the absence of ascorbate ligands required for particle shell formation. If ascorbic acid or dehydroascorbic acid is added to the solution after the NaOH addition and then additional NaOH is added, the solution will turn pale green for the ascorbic acid sample and an amber color for the DHA sample. However, STEM analysis showed that almost all the gold particles did not have an oxide shell (Fig. S11†).

As mentioned earlier, the negative citrate ligand is chemically altered during the addition of HCl, as it protonates and is removed from the gold surface, resulting in copper species being less attracted to the gold particles after the loss of the negative ligand. In both the cycling experiments in Fig. 4 and the attempted cycling experiments of the washed particles above, we do not observe the formation of the oxide shell, as the citrate is removed from the gold surface after interaction with HCl. To demonstrate if this is the case, citrate was added to the washed particles experiment after the first NaOH addition, but before the addition of the ascorbic acid, and the solution was allowed to sit for several minutes. The solution turned green, and STEM analysis confirmed that most of the particles had a copper oxide shell (Fig. S12†). These experiments confirm the importance of the negative citrate ligand on the gold nanoparticles in forming a core–shell morphology and the importance of the ascorbate or dehydroascorbic acid as a copper-bound ligand.

## Conclusions

Gold nanoparticles with a copper oxide shell were synthesized and characterized with electron microscopy, UV-Vis-NIR spectroscopy, and spectrofluorometry. Spherical gold core particles with a 30–40 nm Cu_2_O shell can red shift absorption by approximately 90 nm. Various-shaped gold core particles were used to grow core–shell particles, including nanorods, nanobipyramids, and nanoshells. The gold nanobipyramids with a Cu_2_O shell displayed the largest red shift of 255 nm into the near-infrared. The stability of the oxide shells was studied by cycling the pH of the colloidal solution and observing the rapid dissolution or re-formation of the oxide shells as HCl and NaOH were added to the solution. After several cycles, nanospheres of Cu_2_O, approximately 400 nm in diameter, began to grow. This was a result of the nucleation of Cu_2_O nanocrystals onto other Cu_2_O nanocrystals, instead of nucleation on the gold particles. The importance of the citrate attachment to the gold nanoparticle in forming the oxide shell was demonstrated, as was the presence of a copper-bound ligand in the growth solution.

## Author contributions

Bartolucci contributed to conceptualization, experiments, and writing. Leff contributed to experiments involving TEM and SAED. Maurer contributed to conceptualization, writing and editing. All authors agreed on the final version of the manuscript.

## Conflicts of interest

There are no conflicts to declare.

## Supplementary Material

NA-006-D3NA01000G-s001
