# Supplementary material for: Gold–copper oxide core–shell plasmonic nanoparticles: the effect of pH on shell stability and mechanistic insights into shell formation
Source: Nanoscale Adv. 2024 Apr 15;6(9):2499–507. doi: 10.1039/d3na01000g (PMC11059517; doi:10.1039/d3na01000g)
Supplement: NA-006-D3NA01000G-s001 [file NA-006-D3NA01000G-s001.pdf]

Electronic Supplementary Material (ESI) for Nanoscale Advances.
This journal is © The Royal Society of Chemistry 2024

Gold-Copper Oxide Core-Shell Plasmonic Nanoparticles and the Effect of pH on Shell Stability

Stephen F. Bartolucci, [a] Asher C. Leff, [b] and Joshua A. Maurer [a]*

[1] US Army Combat Capabilities Development Command Armaments Center, Watervliet, NY 12189, United States

[2] US Army Research Directorate, Combat Capabilities Development Command, Army Research Laboratory, Adelphi, MD 20783, United States

[3] General Technical Services, LLC, Wall, NJ 07727, United States

* Corresponding author

SUPPLEMENTAL MATERIAL

Supplemental Figures

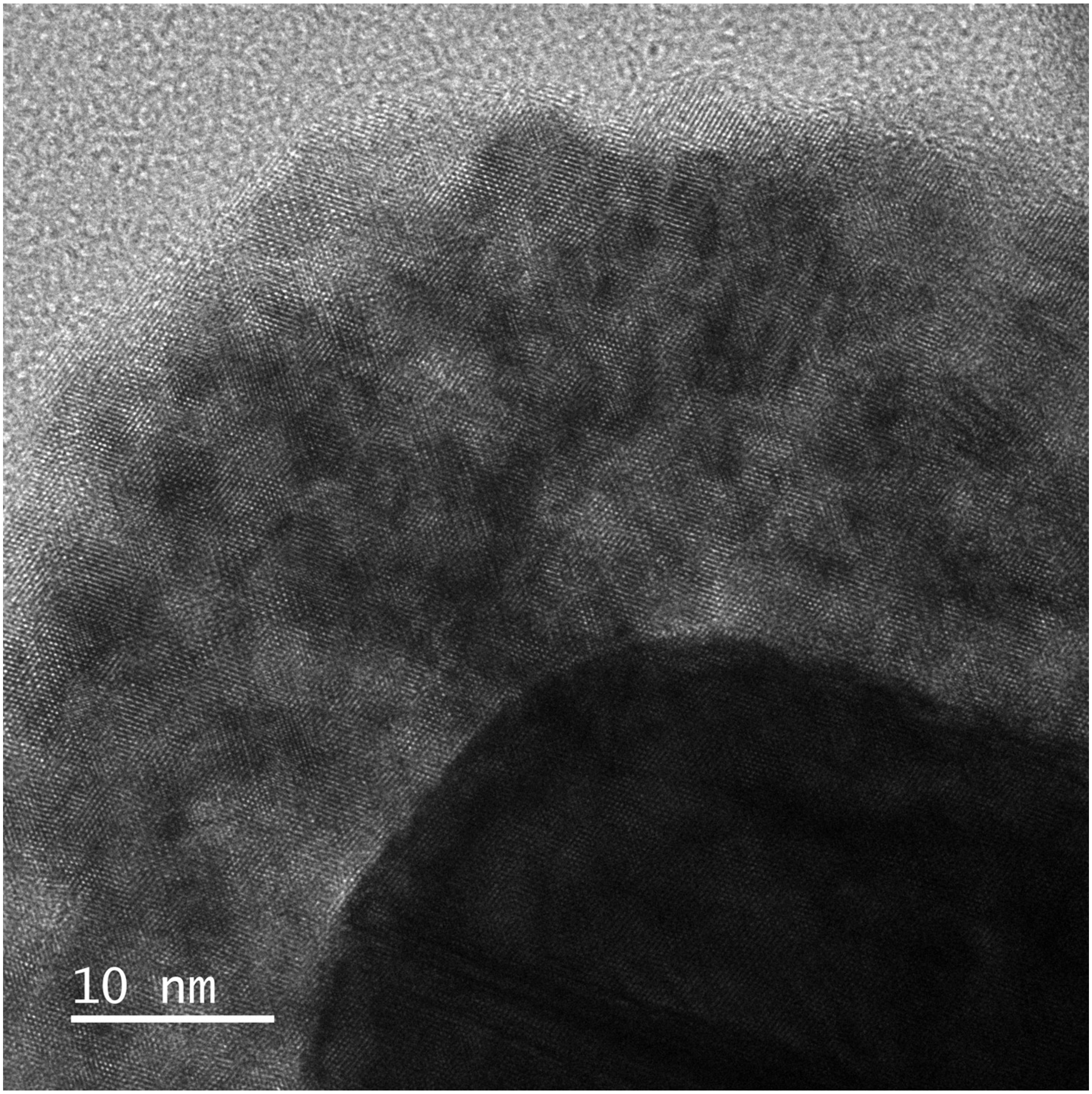

Figure S1. TEM image of spherical 50 nm gold nanoparticle with nanocrystalline Cu_2O shell.

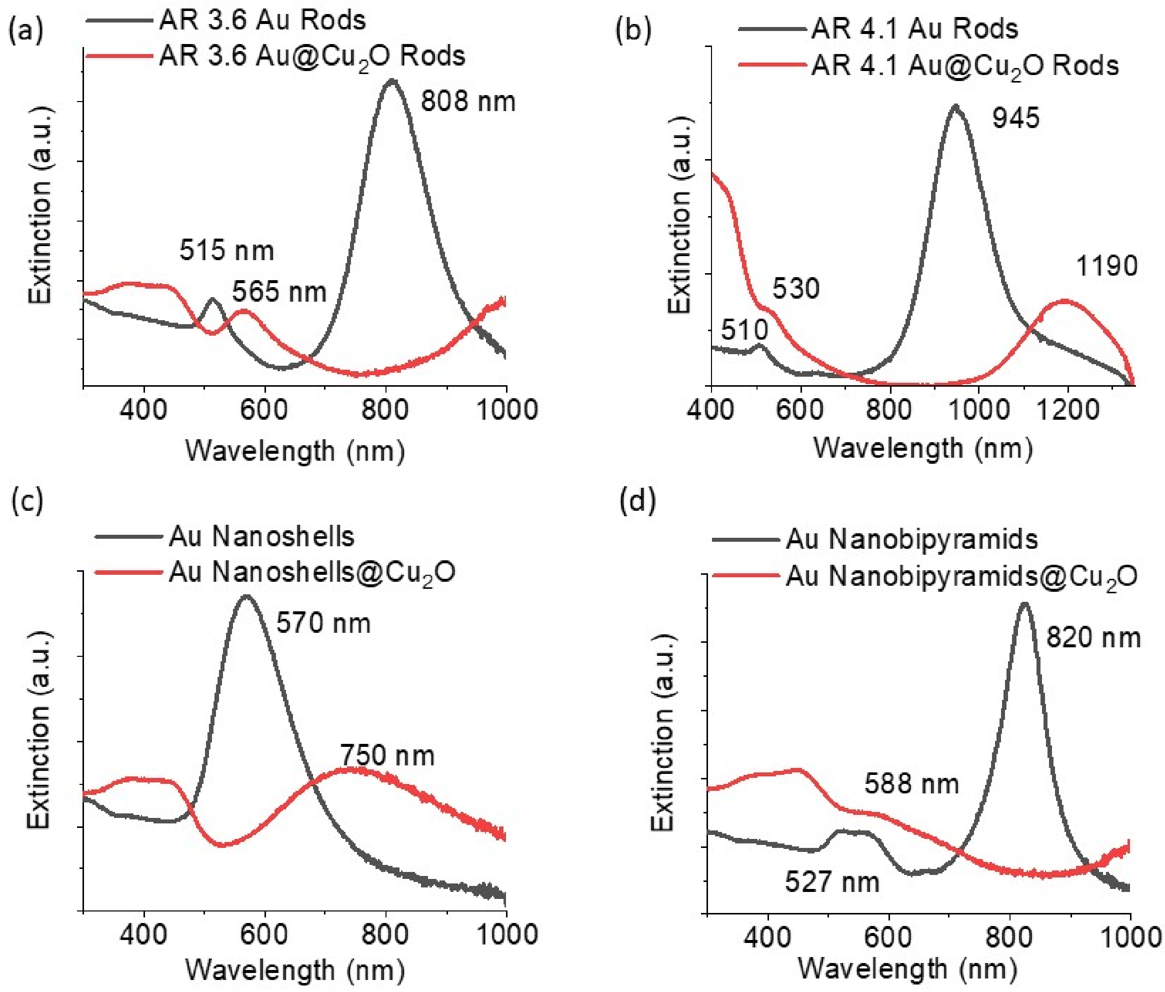

Figure S2. UV-Vis-NIR extinction spectra for gold nanorods (a) A.R. 3.6 (b) A.R. 4.1 (c) gold nanoshells and (d) gold nanobipyramids. Plots are not normalized and show spectra above 350 nm. Transverse plasmon absorption peaks are observed for the nanorods (515 nm for A.R 3.6 and 510 for A.R. 4.1) and nanobipyramids (527 nm), as well as their red-shifted peaks due to Cu_2O shell. In addition, Cu_2O nanocrystal absorption peaks at 385 and 440 nm are seen in each of the plots. (note: data in (b) was only collected down to 400 nm)

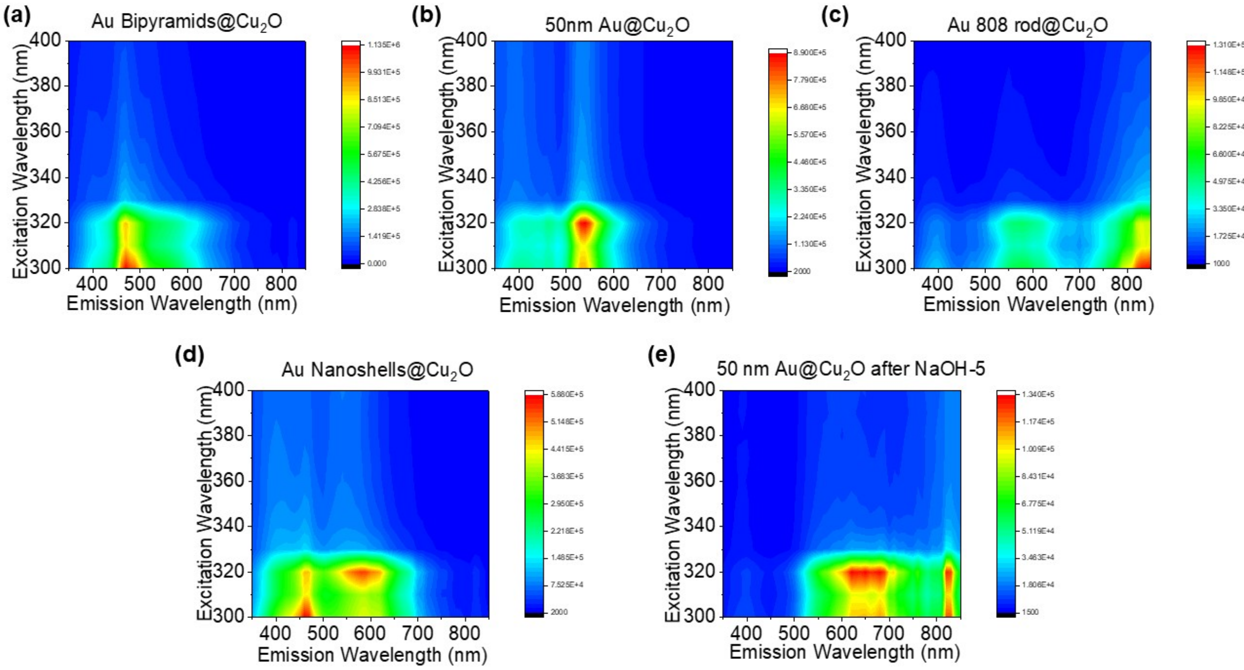

Figure S3. Photoluminescence excitation and emission maps for various core-shell particles. (1) Au bipyramids@Cu_2O (b) 50nm round Au@Cu_2O (c) A.R. = 3.6 Au rods@Cu_2O (d) Au nanoshells@Cu_2O (e) 50 nm round Au-Cu_2O after 5th cycle of NaOH, where solution consists of gold nanoparticles and Cu_2O nanospheres.

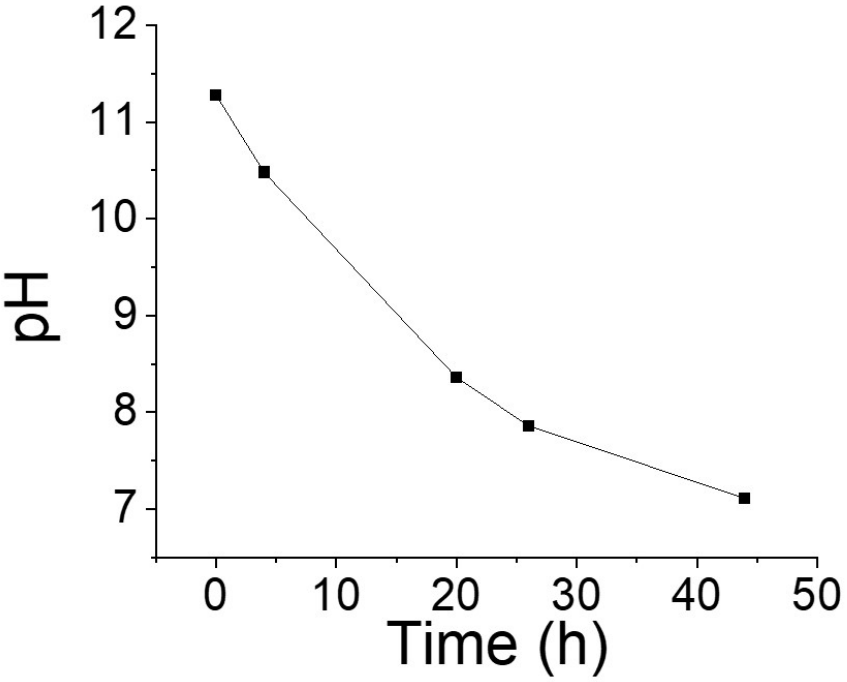

Figure S4. Measured pH of synthesized core-shell particles from start of growth to 44 h after start of growth

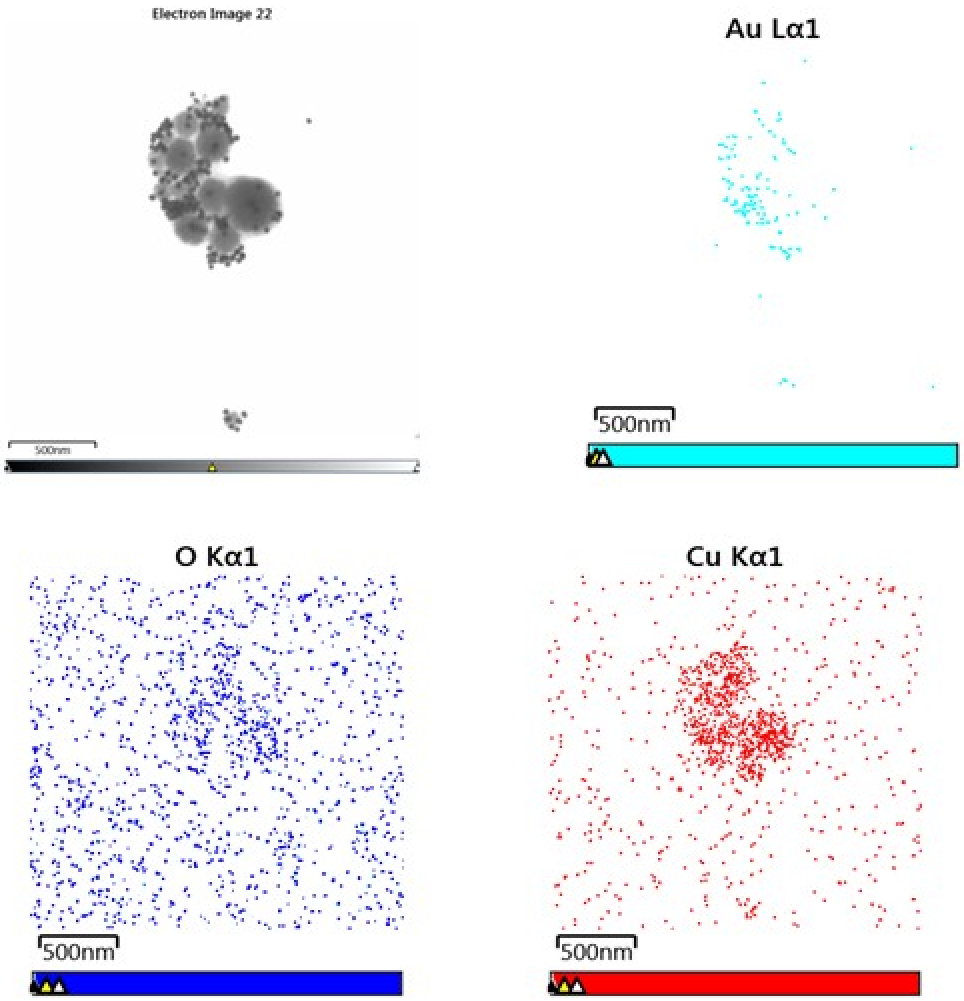

Figure S5. Energy dispersive spectroscopy (EDS) of gold nanoparticles and Cu_2O nanospheres, confirming the presence of Cu, O and Au.

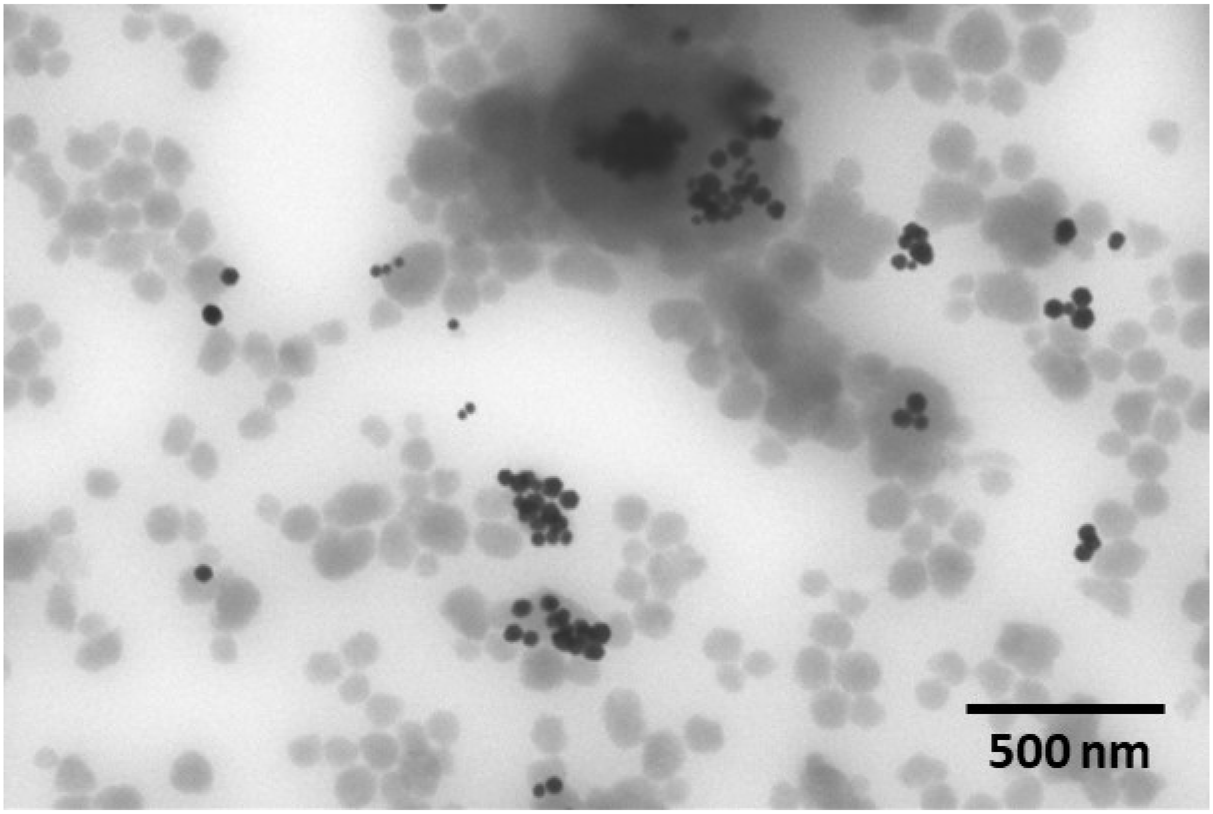

Figure S6. STEM of 1-propanethiol functionalized 50 nm gold nanoparticles used for growth procedure. Core-shell particles did not form, as the copper oxide particles nucleated homogeneously and not on the gold particles.

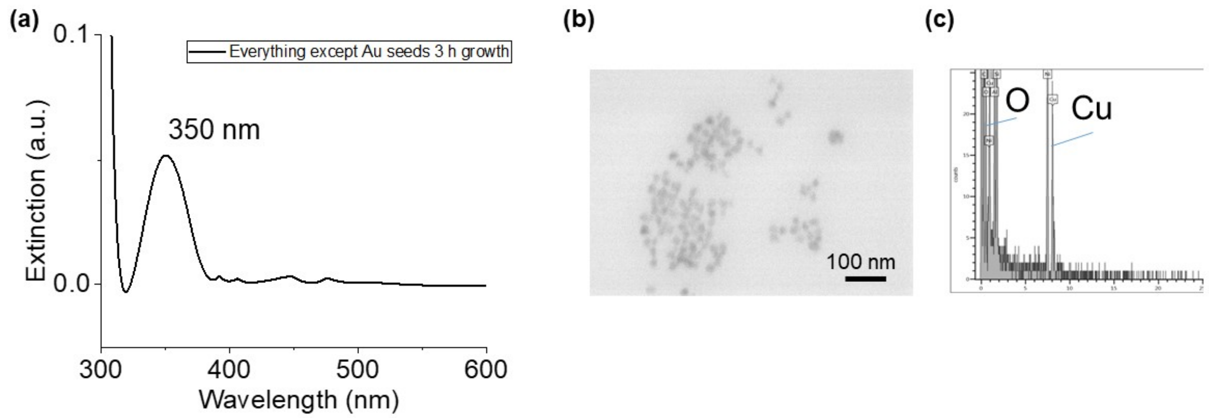

Figure S7. Growth solution with everything except the 50 nm gold seeds after 3 h of growth. An extinction peak appears at 350 nm. (b) STEM analysis shows the formation of 20 nm particles (c) consisting of Cu and O confirmed by EDS.

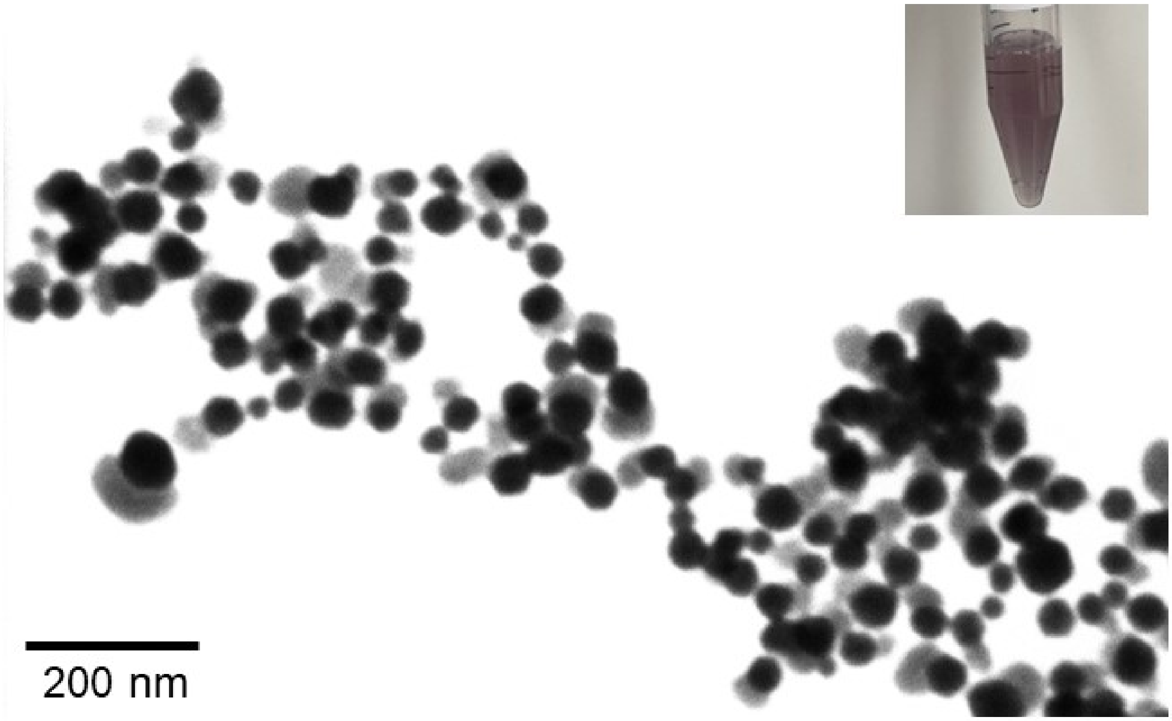

Figure S8. STEM image and vial of purple solution (inset) after 2 minutes of bubbling green core-shell growth solution with CO_2 gas.

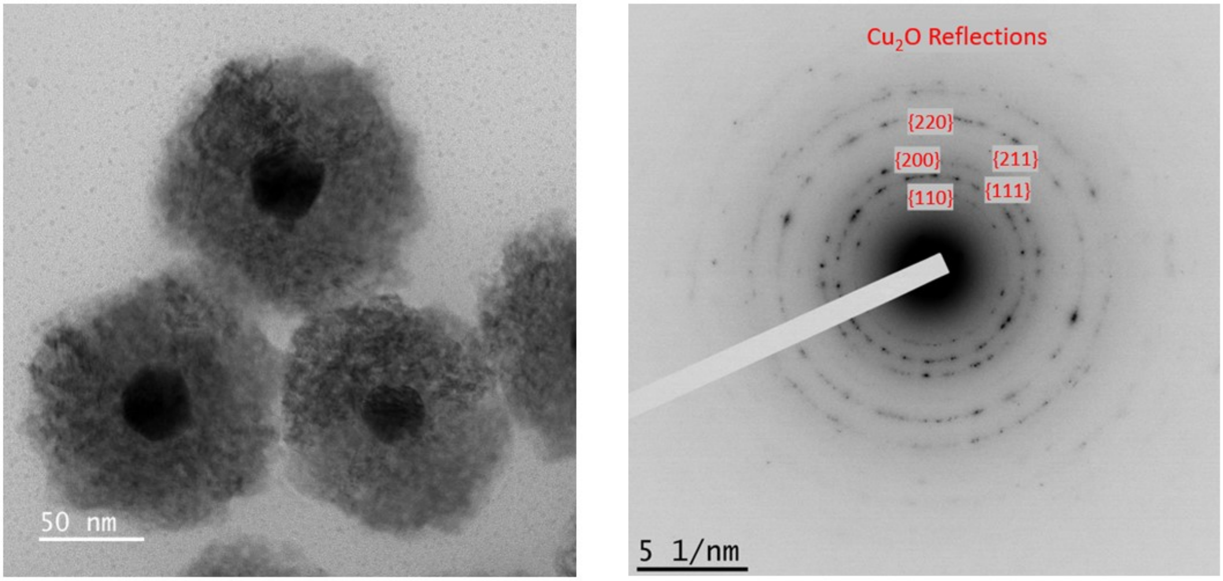

Fig. S9. TEM and SAED of core-shell particles synthesized using CuCl, showing that the shell is Cu_2O.

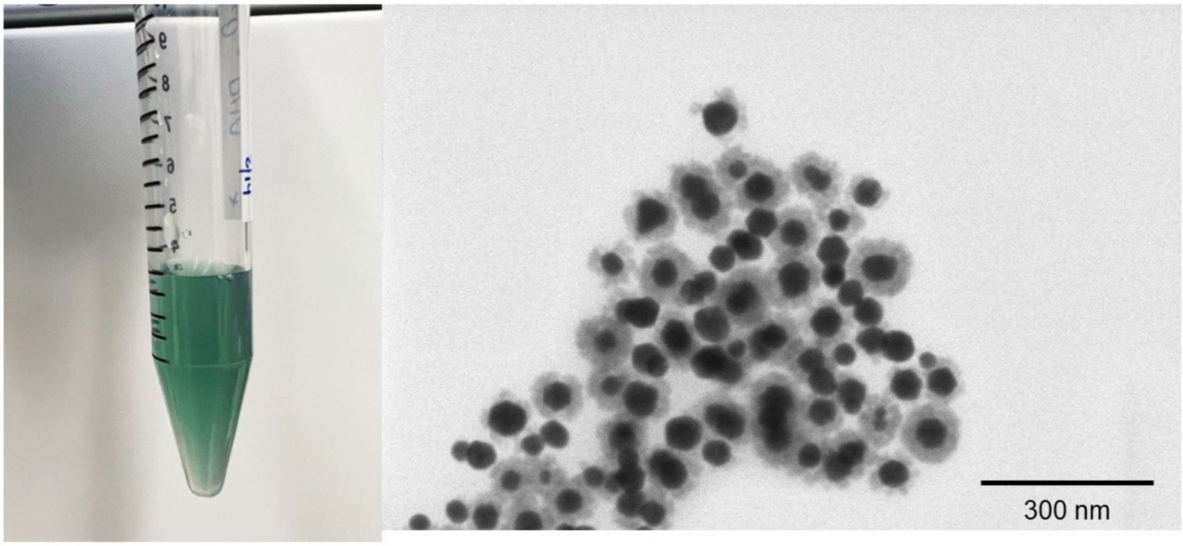

Figure S10. Growth using CuCl and dehydroascorbic acid.

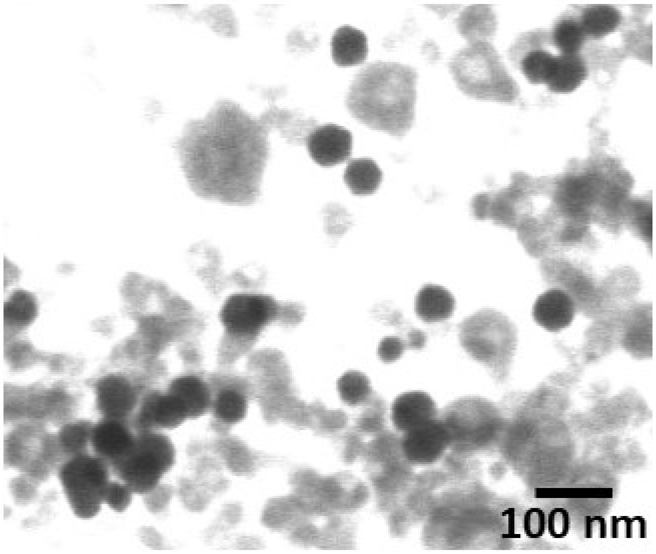

Figure S11. STEM of particles after the addition of ascorbic acid to the washed and cycled (with HCl and NaOH) particles. The dark particles are gold, and the lighter color material is a copper-oxide species.

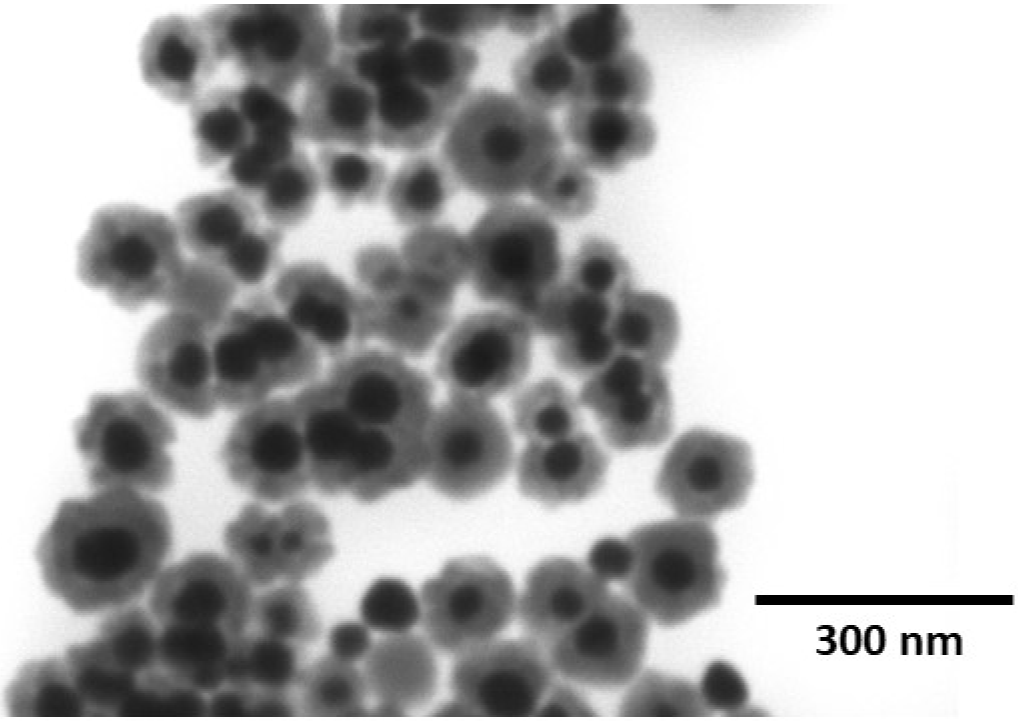

Figure S12. STEM of particles after the addition of citrate and ascorbic acid to the washed and cycled (with HCl and NaOH) particles. The citrate addition results in core-shell particles.

Supplemental Tables

Table S1. Extinction peaks of nanoparticles and core-shell nanoparticles.

**Nanoparticle**	**Oxide thickness (nm)**	**Main Peak (nm)**	**Main Peak with Cu_2O Shell (nm)**	**Dl shift (nm)**
Au Rods (A.R. = 3.6)	32.2 ± 8.5	808 (FWHM = 157)	1045 (FWHM = 233)	237
Au Rods (A.R. = 4.1)	36.9 ± 9.6	945 (FWHM = 159)	1190 (FWHM = 213)	245
Au Nanoshells	34.8 ± 10.3	570 (FWHM = 134)	740 (FWHM = 252)	170
Au Bipyramids	39.7 ± 16.1	820 (FWHM = 110)	1075 (FWHM = 190)	255
Au Spheres	32.0 ± 4.8	533 (FWHM = 56)	622 (FWHM = 110)	89
